# Patients' request for and emergency physicians' prescription of antimicrobial prophylaxis for anthrax during the 2001 bioterrorism-related outbreak

**DOI:** 10.1186/1471-2458-5-2

**Published:** 2005-01-05

**Authors:** Nkuchia M M'ikanatha, Kathleen G Julian, Allen R Kunselman, Robert C Aber, James T Rankin, Ebbing Lautenbach

**Affiliations:** 1 Division of Infectious Disease Epidemiology, Pennsylvania Department of Health, Harrisburg, PA, USA; 2 Division of Infectious Diseases, Penn State Milton S. Hershey Medical Center, Hershey, PA, USA; 3 Department of Health Evaluation Sciences, Penn State Milton S. Hershey Medical Center, Hershey, PA, USA; 4 Division of Infectious Diseases, Department of Medicine, University of Pennsylvania School of Medicine, Philadelphia, PA, USA; 5 Center for Clinical Epidemiology and Biostatistics and Center for Education and Research on Therapeutics, University of Pennsylvania School of Medicine, Philadelphia, PA, USA

## Abstract

**Background:**

Inappropriate use of antibiotics by individuals worried about biological agent exposures during bioterrorism events is an important public health concern. However, little is documented about the extent to which individuals with self-identified risk of anthrax exposure approached physicians for antimicrobial prophylaxis during the 2001 bioterrorism attacks in the United States.

**Methods:**

We conducted a telephone survey of randomly selected members of the Pennsylvania Chapter of the American College of Emergency Physicians to assess patients' request for and emergency physicians' prescription of antimicrobial agents during the 2001 anthrax attacks.

**Results:**

Ninety-seven physicians completed the survey. Sixty-four (66%) respondents had received requests from patients for anthrax prophylaxis; 16 (25%) of these physicians prescribed antibiotics to a total of 23 patients. Ten physicians prescribed ciprofloxacin while 8 physicians prescribed doxycycline.

**Conclusion:**

During the 2001 bioterrorist attacks, the majority of the emergency physicians we surveyed encountered patients who requested anthrax prophylaxis. Public fears may lead to a high demand for antibiotic prophylaxis during bioterrorism events. Elucidation of the relationship between public health response to outbreaks and outcomes would yield insights to ease burden on frontline clinicians and guide strategies to control inappropriate antibiotic allocation during bioterrorist events.

## Background

The September 11, 2001 terrorism events and the ensuing anthrax attacks in the United States were associated with widespread psychological trauma [1]. In response to the outbreak, public health officials disseminated recommendations for *Bacillus anthracis *postexposure prophylaxis and clinical management of patients with anthrax disease [2,3]. For asymptomatic persons, antimicrobial prophylaxis was indicated only for confirmed or suspected aerosol anthrax exposure documented by public health or law enforcement [4-6].

Public health officials assured the public that emergency drug supplies would be delivered to clinical settings as needed from the national antimicrobial stockpile [7]. Despite such assurances and caution against personal stockpiling and self-medication, media reports of increased demand for ciprofloxacin indicated a potential public health problem [8]. However, it was not clear to what extent individuals with self-identified risk of anthrax exposure approached physicians for antimicrobial prophylaxis. Additionally, the response of frontline clinicians to these requests had not been described. To address these questions, we used data from a large public health survey of Pennsylvania emergency physicians following the 2001 terrorist attacks [9].

## Methods

A total of 250 potential study subjects were randomly selected from the 2001 membership database (n = 1,060) of the Pennsylvania Chapter of the American College of Emergency Physicians (ACEP). Information in the database, such as contact telephone numbers and location of practice, is provided during enrollment or during membership renewals, and no active verification is done. Because the accuracy of the database was unknown, we used an 80% estimate based on a previous study of emergency physicians [10]. During November 2001-January 2002, we conducted a telephone survey with items designed to assess requests for and prescriptions of antibiotic prophylaxis for anthrax. For the types of antibiotics prescribed, the survey choices were ciprofloxacin, doxycycline, amoxicillin, penicillin VK, other, or none. Emergency departments with post-graduate training programs were categorized as "academic" while all others were categorized as "non-academic." Details concerning survey instrument design and data collection are described elsewhere [9]. Population and administrative data maintained by the Pennsylvania Department of Health's Bureau of Health Statistics were used to stratify respondents by geographic location. Counties with a population density of ≥ 450 persons per square mile were defined as "urban" while all others were categorized as "rural." "Eastern" Pennsylvania was defined as the Southeast and Northeast Districts of the Pennsylvania Department of Health. All other locations were considered "western."

We used one-way frequency analyses to describe distributions of responses for all categorical items. Associations were quantified using odds ratios (ORs) with associated 95% confidence intervals (CIs). Statistical analyses were performed using SAS software (SAS Institute Inc., Cary, NC).

## Results

Forty-three of the 250 physicians in the sample were excluded from the study because of insufficient contact information or because the physician was no longer practicing medicine in the state. Of the remaining 207 physicians (24% of the estimated population of 848 subjects with accurate information), 97 were interviewed (47% response rate).

Sixty-four (66%) of the 97 respondents had received patient requests for antimicrobial prophylaxis against anthrax; of the physicians who received requests, 16 (25%) prescribed antibiotics. Physician setting (urban vs. rural; eastern vs. western) was not associated with either patient request for, or physician prescription of, antimicrobial prophylaxis (Table 1). Of the 52 respondents in urban areas, 38 (73%) had received requests for antibiotics while 26 (59%) respondents in rural counties had received such requests (odds ratio [OR], 1.9; 95% confidence interval [CI], 0.8 – 4.4). Similarly, the type of institution (academic versus non-academic) was not associated with requests for or prescriptions of antibiotics.

**Table 1:** Physicians' response to patients' requests for anthrax prophylaxis. *

	**Characteristic**								
	Setting no (%)^†^			State location no (%)^‡^			Emergency department type no (%)^§^		

**Variable**	Urban	Rural	OR (95% CI)	Eastern	Western	OR (95% CI)	Academic	Non academic	OR (95% CI)

Received requests for antibiotics	38 (73)	26 (59)	1.9 (0.8–4.4)	33 (70)	31 (63)	1.4 (0.6–3.2)	21 (68)	41 (66)	1.1 (0.4–2.7)
Prescribed antibiotics for anthrax	10 (20)	6 (14)	1.5 (0.5–4.7)	6 (13)	10 (21)	0.6 (0.2–1.8)	7 (23)	9 (15)	1.8 (0.6–5.4)
Requested testing for anthrax	18 (35)	11 (24)	1.6 (0.7–4.0)	15 (32)	14 (28)	1.2 (0.5–2.9)	8 (26)	21 (33)	0.7 (0.3–1.8)

Abbreviations: OR: Odds ratio; CI: Confidence interval.

* The ninety-seven survey respondents were used in the analysis.

^†^Fifty-two physicians responded from practices located in one of the eleven "urban" counties that had a population density of ≥450 persons per sq mile in 2001.

^‡^Forty-seven respondents were from eastern Pennsylvania, defined as the Northeast and Southeast Districts.

^§^Thirty-one physicians responded from practices that were considered "academic" based on presence of training for residents or fellows.

**Figure 1:**
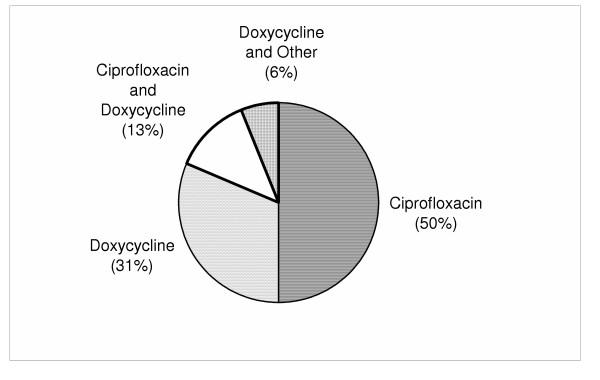
**Types of antibiotics prescribed by emergency physicians. **A total of 16 physicians prescribed various antibiotics shown above. Fifteen physicians prescribed these types of drugs to 23 patients. One physician did not respond to the question on the number of patients the physician had given anthrax prophylaxis.

Fifteen physicians prescribed antibiotic prophylaxis to 23 patients; nine physicians prescribed to one patient, four prescribed to two patients, and two physicians prescribed to three patients. One physician did not answer the question regarding number of patients prescribed prophylactic antibiotics. Ten physicians (63%) prescribed ciprofloxacin while 8 physicians (50%) prescribed doxycycline (Figure 1). Fourteen (88%) of the respondents that prescribed antibiotics to patients also referred the patients for diagnostic tests for anthrax exposure.

## Discussion

Following the September 11 terrorist attacks and the ensuing anthrax outbreak, the majority of the emergency physicians we surveyed in Pennsylvania had received patient requests for anthrax prophylaxis; a quarter of these physicians prescribed antibiotics for these patients. Physicians that reported patients' requests for antimicrobial prophylaxis were distributed across the state, suggesting that patients' search for protection against anthrax was widespread. Our results are consistent with data on specimens submitted to Pennsylvania public health laboratory officials for *B. anthracis *analysis. During October-December 2001, the Pennsylvania Bureau of Laboratories received approximately 1400 specimens including white powder, environmental swabs, and letters from counties in eastern and western Pennsylvania districts. Of these, 27 (or about 18 requests per 100,000 population) came from Cambria County in the western district (PA DOH: unpublished data). There were 11,063 anthrax-related telephone inquiries received from October 8 to November 11, 2001 by the Centers for Disease Control and Prevention's (CDC) emergency operations centers; queries originated from all states and one US territory. Most of these calls were from members of the public concerning anthrax vaccines (≈58%), suggesting that search for protective measures against anthrax was widespread across the United States [11].

Other studies have documented an increased use of antimicrobial agents that was temporally related to the anthrax outbreak; this use could not be ascribed to that recommended by the CDC. For example, a recent national study reported that approximately 160,000 more ciprofloxacin and 96,000 more doxycycline prescriptions were written in 2001 compared to 2000 [12]. When other investigators compared ciprofloxacin utilization in 2001 with 2000, they found that it declined for all months except October 2001 when ciprofloxacin utilization increased 9.8%. They also found that the increase was not limited to areas where anthrax cases had occurred, suggesting that many Americans sought antibiotic prophylaxis [13].

Of the physicians reporting that they had prescribed antibiotics for anthrax prophylaxis, the majority used ciprofloxacin. This finding is consistent with other studies [12,13] and is likely because the initial CDC guidelines recommended ciprofloxacin prophylaxis for *B. anthracis *exposure until susceptibility results were known [4,5]. When tests showed that *B. anthracis *isolates recovered from patients involved in the anthrax attacks were susceptible to other antibiotics, public health officials indicated that doxycycline might be preferable over ciprofloxacin [2]. While both drugs are approved for postexposure prophylaxis [14], the rationale for favoring doxycycline was to prevent ciprofloxacin resistance in more common bacteria.

Unfortunately use of antibiotics has inherent risks and costs and optimizing benefits is especially difficult in the midst of bioterrorist events. Consequences of antibiotic treatment of unexposed individuals include adverse drug reactions, increased risk of antimicrobial resistance, depletion of antibiotics, and monetary costs [15]. Furthermore, use of emergency departments for sporadic distribution of prophylactic antibiotics to persons presenting with self-identified risk appears inefficient. It is unclear whether these persons can be adequately managed in emergency departments without the support of public health and law enforcement officials.

Public health response likely influences demand for and outcomes associated with antibiotics requests during bioterrorism attacks. When we asked physicians to suggest on what health departments could do to reduce the influx of patients to the emergency departments, they cited official communications to make the public "less worried." In Illinois, a surge in environmental samples received by public health officials for anthrax tests was associated with both media reports of anthrax cases in other states and a specific announcement on October 29, 2001 by the US attorney general and the FBI director. The announcement asked US citizens and law enforcement agencies to be on the "highest alert" based on "credible information" [16]. Lessons learned from the 2001 anthrax attacks in New Jersey suggest that communities in which the public health sector and clinicians have a strong working relationship are better prepared to meet mass prophylaxis needs [17]. Similarly, lessons learned in New York City during the same outbreak demonstrated the benefits of advance logistical planning for mass postexposure prophylaxis including an antibiotics distribution site and clear eligibility criteria [18].

We acknowledge some limitations to our results. First, as in any survey, these data are subject to non-response bias. But the 47% response rate is comparable with other telephone surveys conducted among physicians in general [19]. In addition, responders and non-responders had similar baseline characteristics, suggesting that these groups were comparable [9]. Second, the study is limited to types of antibiotics prescribed and cannot be used to estimate dosage, number of pills allotted to these patients, costs, or compliance to treatment for perceived or real anthrax exposure. Third, the indications for prophylaxis were not studied. While it is not certain, it is likely that at least the vast majority of the antibiotic perscriptions found in this study were outside indications described in public health guidelines. It is plausible that some patients sought prescriptions for storage; it is also likely that they at least initiated the antibiotic course.

## Conclusions

Taken together with other recent studies of antibiotic utilization [12,13], these data suggest a need for public health response to increased demand for prophylaxis against perceived bioterrorism exposures. Experiences in areas where mass prophylaxis was delivered and suggestions made by the respondents in this study suggest that improved public health communications might reduce the influx of patients to emergency departments. In addition, as demonstrated by New York City's exemplary response to the West Nile virus outbreak in 1999 and the 2001 anthrax attacks, prior logistical plans and strong working relationships among public health officials and clinicians are essential [18,20]. Elucidation of the relationship between public health response to outbreaks and outcomes may offer lessons to reduce inappropriate demand for and prescriptions of antibiotics during bioterrorist events.

## List of abbreviations

ACEP: American College of Emergency Physicians

CDC: Centers for Disease Control and Prevention

PA DOH: Pennsylvania Department of Health

## Competing interests

The author(s) declare that they have no competing interests.

## Authors' contributions

NMM conceived of the study and its design, and coordinated revision of the manuscript drafts with all authors. KGJ participated in data collection, study design, and contributed to the study's critical review. ARK participated in the design of the study and performed statistical analyses. RCA secured funding and participated in the study's critical review. JTR participated in the study design and coordination. EL contributed to the design of the study and participated in its critical review. All authors read, commented on, and approved of the final manuscript.

## Pre-publication history

The pre-publication history for this paper can be accessed here:


